# Regenerative Food Innovation: The Role of Agro-Food Chain By-Products and Plant Origin Food to Obtain High-Value-Added Foods

**DOI:** 10.3390/foods13030427

**Published:** 2024-01-28

**Authors:** Charles Stephen Brennan

**Affiliations:** School of Science, RMIT University, Melbourne, VIC 3000, Australia; charles.brennan@rmit.edu.au

**Keywords:** regeneration, food innovation, sustainability, biomolecules, nutrition, wellbeing, valorisation

## Abstract

Food losses in the agri-food sector have been estimated as representing between 30 and 80% of overall yield. The agro-food sector has a responsibility to work towards achieving FAO sustainable goals and global initiatives on responding to many issues, including climate pressures from changes we are experiencing globally. Regenerative agriculture has been discussed for many years in terms of improving our land and water. What we now need is a focus on the ability to transform innovation within the food production and process systems to address the needs of society in the fundamental arenas of food, health and wellbeing in a sustainable world. Thus, regenerative food innovation presents an opportunity to evaluate by-products from the agriculture and food industries to utilise these waste streams to minimise the global effects of food waste. The mini-review article aims to illustrate advancements in the valorisation of foods from some of the most recent publications published by peer-reviewed journals during the last 4–5 years. The focus will be applied to plant-based valorised food products and how these can be utilised to improve food nutritional components, texture, sensory and consumer perception to develop the foods for the future.

## 1. Food Waste and Sustainability Impact

**Figure 1:**
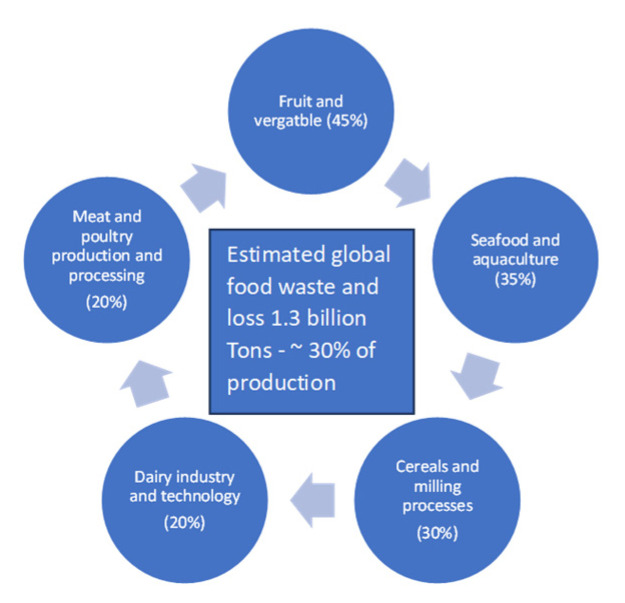
Pictorial representation of total amount of estimated food waste and loss per year based on the primary sectors.

Food losses due to the processing and production operations of the agri-food sector have been estimated to represent between 30 and 80% of overall yield [1], and this can be up to 1.3 billion metric tons of food material wasted each year, as illustrated in Figure 1. The majority of the losses have been calculated as originating from waste issues in the fruit and vegetable industry (possibly due to the high perishability of such products). However, the aqua food industry and meat industries closely follow the overall amount of production and processing loss related to supply chain issues [2].

### 1.1. Distribution of Origins of Food Waste and Loss across the Globe

Many researchers have illustrated that this issue of waste material originates from processing, supply chain and consumer use stages of the lifecycle of food rather than production losses specifically derived from farming practices [2]. This is also dependent on culture and governmental influence. For instance, in Australasia, the amount of food loss from grocery supply chains accounts for 5–6% of all food loss, whereas in Europe, the figure is close to 16%, whilst in Asia, this can be between 20 and 30% [3]. The research from Martindale et al. [2] illustrates the need for an integrated approach to achieve the goals of sustainability, which we have been addressing for a while, and that this needs to reflect strategies to reduce the impact of climate change on the supply and security of the food and beverage industry. Table 1 illustrates a recent estimate of global food waste produced on behalf of each household in major countries throughout the world. What it illustrates is that there is a significant variation in the estimated food waste per capita each year depending on regionality and supply chain procedures [3]. What is of interest is the per capita differentiation between countries, possibly due to cultural influence. 

### 1.2. Potential of Digital Technology in Guiding Us through Process Optimisation

The development of the Internet of Things (IoT) will aid the digitalisation of the food processing and production sectors, and understanding the data that is within such studies would help improve our insight into the potential methodologies we can employ to reduce waste as well as optimise industry processing. For instance, Caldeira et al. [4] approached the issue from a mass balance exercise across multiple stages of the lifecycle analysis within the food industries in Europe and broke the potential of waste generation into specific areas across the lifecycle of food. Such a high level of waste has been subjected to evaluation in relation to the FAO sustainable goals and global initiatives in harnessing food ingredients from secondary side streams of the agri-food industry [5]. The focus of the analysis by Caldeira et al. [4] was on the opportunity to develop strategies that could address Sustainable Development Goal 12, which emphasises ensuring sustainable consumption and production patterns across society and which has led to governmental policy documents outlining a proposal to reduce levels of waste by up to a half by 2030. 

### 1.3. Sustainability, Food Waste and Environmental Concerns on Food Valorisation

**Figure 2:**
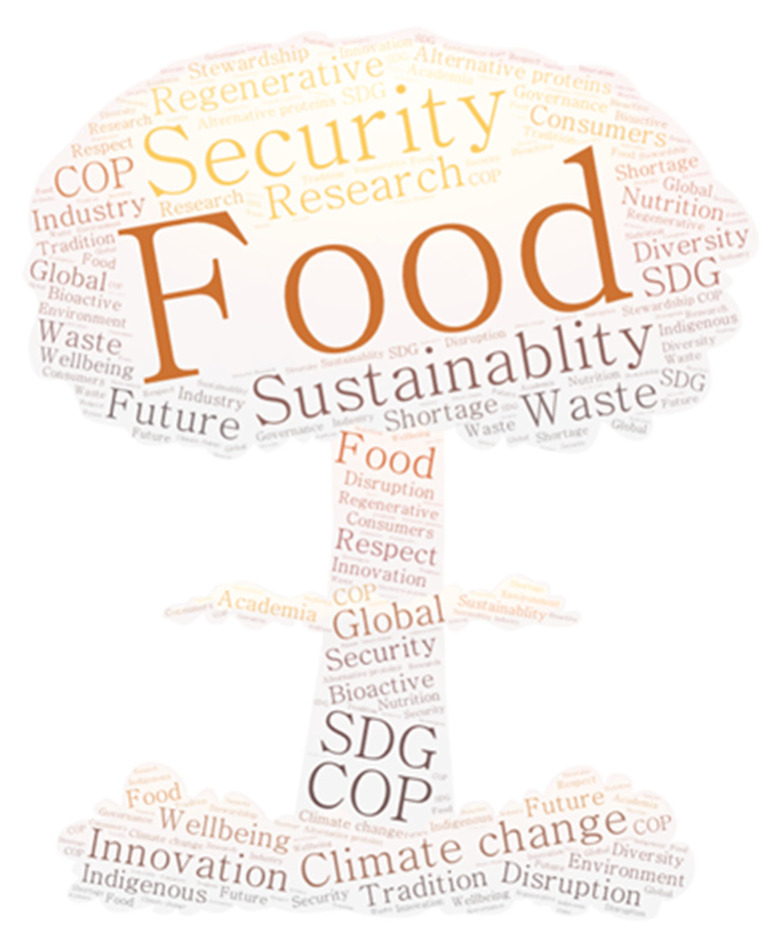
A simplified pictorial of the independent components that interact when considering food sustainability and security. In particular, there is a dependency on supply chain, processing and production systems in reducing waste generation in order to protect the environment for the future.

Figure 2 is a simplistic diagrammatic representation of the complexity of the factors involved in food waste utilisation in the food industry. Researchers have identified that food waste quantities vary across sectors and regions, with differences in practices, infrastructure and cultural norms impacting the amount of waste generated. 

The environmental impact of food waste is considered a high priority from a governmental policy framework as it contributes to greenhouse gas emissions, as decomposing food releases methane in landfills. The FAO has reported that the carbon footprint of food waste could be as much as 3.3 billion tons of carbon a year, having a significant effect on the levers of climate change [5]. The impact of high levels of food waste on sustainability and resource use cannot be underestimated, especially in the food production and processing industries, which consume resources including water, land and energy. As such, this waste production exerts economic implications as loss of resources invested in production, transportation and distribution. It affects profitability for businesses and can lead to increased costs for consumers as well as food scarcity [3,4].

So, while the wasting of foods across the supply chain raises ethical concerns in a world where millions of the population face hunger, it remains a possibility that redirecting food waste to those in need can address some aspects of food insecurity issues. This emphasis on employing measures to reduce waste production in a short period of time has also created intense interest in the opportunity to recover and reuse waste from production and process operations [6,7,8,9]. Much of the focus has been on improving the recovery rates from plant- [10] and meat-based material [11] to reduce the impact on the environment and create added-value products that can be used as ingredients in the circular economy within food ingredient utilisation. These initiatives have a beneficial effect in terms of recovering potentially powerful bioactive compounds, which may be effective in the improvement of human health, with research illustrating the potential to utilise waste streams from plant-based products as enhancers to the nutritional quality of processed foods [9,11,12,13,14].

It is undeniable that as the growth in the world’s population steadily progresses toward the figure of 9 billion individuals, the stress and strain applied to the food production and processing systems upon a delicate global environmental fabric will need to be addressed rapidly [2,4,5]. Reflecting on this from a purely academic viewpoint, we, as thought leaders, have a responsibility to act as stewards for the future and develop a sense of governance of our land and waters, together with food systems regionally, nationally and globally. A word that springs to mind when considering this situation is that of curation. We have the duty to be mature curators of our future whereby, being respectful of the past, we can establish a new vision for the future with the principles of stewardship and governance securely at the centre of our decision-making processes. 

The way that we move forward in the integration of technology, industry and systems will create the impact that is essential for us to fulfil the ambitious sustainable development goals that are highlighted by so many academics and policy makers. Part of this move will be towards understanding geopolitical and human-centred activities, as many of these are so important in directing our attention to how we reinvent the future. Part of this is also how we can apply meaningful technological innovation alongside advances in the comprehension of the role of big data and Industry 4.0 and 5.0 to respond to the opportunity of regenerative food innovation systems [15]. 

### 1.4. Can Valorisation Be the Answer to Sustainability and Regeneration

**Figure 3:**
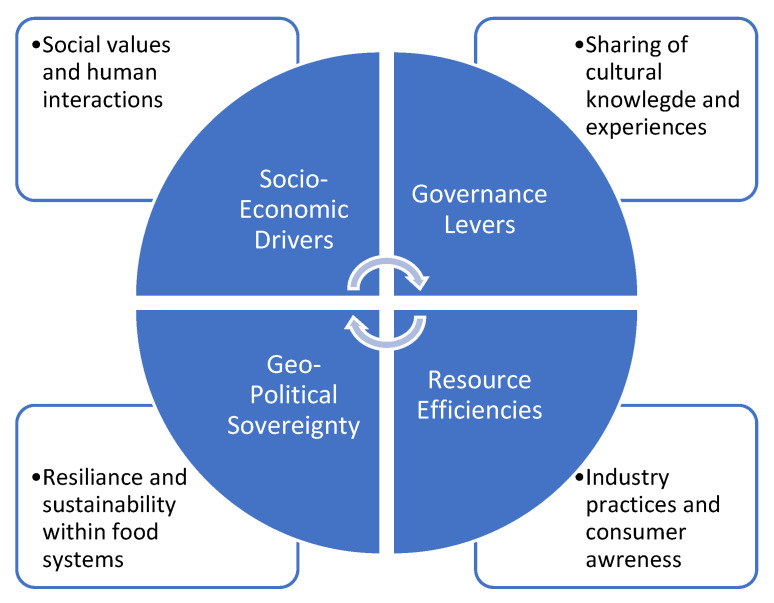
Simplistic representation of factors interplaying with the socioeconomic and geopolitical cross-cultural drivers for regenerative food systems.

This could include the role of waste recovery and reutilisation to address issues of sustainability and food insecurity, with researchers indicating that up to 2% of global food consumers are facing real-life food insecurity issues, which means that up to 2 billion people are witnessing the effects of malnutrition [16]. The signposts are clear and evident for all of us reading academic literature as well as social media content, namely that things have to change. The term regenerative food innovation attempts to create a vision whereby researchers, policy makers, industry stakeholders and the global consumer body can move not only to return to what was considered appropriate in sustainability and security systems of the last decade or two but to reinvent these systems to create newness and a more sustainable and secure future for food production, consumption and innovation. Tittonell et al. [17] recently reviewed the importance of regenerative agriculture in providing agroecological solutions to sustainability across diverse cultures and governments. The authors highlight that the proponents of regenerative agriculture aim for outcomes beyond what sustainability can provide by establishing a balance between agricultural use and practices of the land, which is deeply connected to the concept of governance of land and areas. This could be a mixture of cultural–historical practices, socioeconomic pressures and sovereignty of lands and waters in a complex interconnection of themes (Figure 3). 

There is undoubtable a link here between understanding production practices and process operations in order to achieve some of the future benefits we are seeking. For instance, Das et al. [18] have evaluated the impact of food production and storage processes in order to enhance both food security and sustainability in emerging nations. A good example of this is the recent study that examined the effects of climate change and maritime security in the Indo-pacific regions, as this has direct effects on smaller nations where land-based agricultural systems are only part of the story for sustainability and security issues, as they rely heavily on maritime resources for trade, economic development and basic provision of foods [19]. At the same time, Bhatkar et al. [8,9] illustrated that concerns related to production, processing and post farm gate systems are essential in creating efficiencies in the overall supply chain of food production. Careful consideration of each step in the food production and processing cycle will illustrate potential savings to be obtained at the pre-farm gate stages, harvesting and preparation, ingredient generation and food production operations, as well as cold chain systems and consumer utilisation. Small gains from each of these stages, when combined, could create large wholescale benefits in terms of sustainability savings. Indeed, supply chain dynamics and improving the resilience of blockchains are major subjects in addressing the sustainable development goals placed on food systems [20,21]. 

## 2. Consumers and Their Focus

At the same time, we need to focus squarely on the consumer without wholescale take up from consumers. Any of the advances we achieve through enhancements in production and processing units of operations and in cleaner processes that conserve energy and address the reclamation of waste products will not create an impact in terms of global sustainability and security. It is evident that consumers are increasingly prioritizing sustainability and environmental impact when considering their choice of food products and that foods made from waste streams align with these values by reducing waste and utilizing resources efficiently.

### 2.1. Consumer Perception and Food Valorisation

Consumer perception of food materials recovered from waste streams often revolves around the safety and nutritional quality of foods made from waste. There are concerns about hygiene and the potential presence of contaminants in valorised food materials, which need addressing. Similarly, there is hesitation among consumers to use valorised food ingredients from a taste and textural perception of these foods. The recent research evaluating the perspective of repurposing food waste streams in the Netherlands throws light on the behavioural practices of the actors involved in promoting and applying regenerative food systems to recapture redundant food materials post-food loss and waste [22]. What was interesting about this article was the interview with key participants in the process, highlighting that repurposing food waste attracts great appeal if carried out on a local basis and that this diminished the distance between the source of valorised food products and reutilization products. There is a lot of research that is required to determine this exact relationship in terms of the eyes of the consumer and the perception of improving the local vs. the global waste problems. 

### 2.2. Levers of Adaptation Related to Consumer Taste and Texture

To understand the levers that contribute to consumer perception of repurposing food materials into food products, researchers have endeavoured to tackle the issues around the consumption of alternative food products, whether in unusual tastes, appearances or the actual labelling of the products. For instance, Crawford, Low and Newman [23] examined the specific issue of understanding the barriers that consumers, in this case children, put in place to avoid eating unfamiliar foods. From a psychological viewpoint, this research is fascinating as it explores conceptual aspects of triggers to sensory aversion, which is central to consumer preference and acceptance of foods. The psychological barrier to consumer engagement is the reluctance to try new foods that either look or smell unappealing at first glance. It is not only the possibility of new foods from reclaimed sources of ingredients that may cause consumer hesitancy, but the use of nontraditional plant-based sources of proteins can prove hard for the consumers to engage with, as the study of Munialo [24] illustrated when evaluating different forms of plant protein sources. The impact of animal husbandry on climate change and the sustainability of food systems is called into question a lot; however, there are drawbacks to an over-reliance on arable cropping systems. For instance, whilst plant-based foods are often rich in proteins [9,24], one of the challenges for food researchers and the industry as a whole is how to understand the potential of these ingredients in modern food factory applications where the consistency of ingredients is imperative in order to maintain efficiency and reduce production loss. The study of Munialo [24] explored these issues in relation to the extraction of proteins and the requirement to characterise the functionality of these ingredients in order for them to be used in commonly consumed food products from both a consumer and a cultural identity. This is something that requires further research as we progress with the concepts of utilising novel food materials.

### 2.3. Examples of the Application of Valorised Material into Food Produce

A good example of using recovered by-products in food systems is the consumer view of basic food items such as bread and what is culturally perceived as the norm, especially in relation to the health concerns raised from the excessive consumption of starchy foods such as breads, biscuits and extruded snack products. For instance, from a nutrition and wellbeing viewpoint, the use of whole grains in bread (and other cereal products) has a tremendous advantage in terms of their nutritional diversity and the impact that the range of bioactive ingredients, from whole-grain material (phenolic compounds and enhanced fibre content, to name just two main components) can exert on overall health outcomes of an individual compared to excessively refined flours and the products made with these [25]. Whilst the high composition of dietary fibre and phenolic compounds are of great significance in enhancing human nutrition, the taste and texture of fibre-rich foods are not universally accepted, as too are the flavours that are generated from phenolic-rich ingredients (tending to be bitter due to their high phenolic content). This relates to consumer perceptions of novel or different foods, as previously discussed in the article from Crawford, Low and Newman [23], and whilst that may have had a focus on perceptions of children to different food products, consumer perceptions of foods tend to be formed relatively early in our development. Ross [26] cleverly illustrated the significance of food structure in consumption behaviour when examining the impact of variations in food texture on the perceived ability of individuals to masticate and, hence, enjoy foods. In this work, Ross [26] delved into the importance of adjusting food textures to suit the physiological requirements of consumers using swallowing-compromised cohorts and relating this to the ability to consume. The work, although based on select cohorts, has ramifications across all consumer appreciation of foods (with regard to texture, mouthfeel and enjoyment) and should be considered when evaluating barriers to the consumption of valorised food by-products in the food industry. 

One of the ways to overcome consumer hesitation to engage in the consumption of food waste materials is to evaluate their potential as ingredients of the future. Providing clear and accurate information about the source of ingredients, production processes and safety measures is crucial in influencing consumer perception of the benefits of such ingredients, not only in terms of global sustainability but also in advancing human nutrition. A plethora of research has focused on how some waste streams can be adapted in order to capture the bioactive compounds within by-products and upcycle these as ingredients to supply added nutritional benefits [27,28,29]. Extraction processes have come under intense research for a number of reasons, which include the ease of extraction of the beneficial bioactive ingredients, the thermal and enzymic stability of the molecular structure of the recovered compounds, and also the drive for a clean label ingredient and hence the requirement for what we can collectively call green technology for their retrieval [30,31,32,33]. From a traditional perspective, the most common ways of recovering bioactive fractions from agricultural waste products have tended to be based on the use of organic solvents such as methanol, ethanol or acetone. The mining of waste streams for potentially valuable ingredients that could be used in the food industry has been central to the idea of value-added commodities from valorisation. Melini et al. [34] illustrated that the recovery of phenolic compounds from agricultural by-products could be valuable in creating nutrient ingredients to be incorporated into foods such as bread and enhance the antioxidant levels of the product, thus creating a potential benefit for the consumer in terms of nutritional profiles. In a similar vein, Nunes et al. [35] illustrated the potential of recovering by-products from the olive oil industry as therapeutic ingredients. We understand that olive oil has numerous benefits as a food ingredient and as part of the Mediterranean diet, associated with exceptional lifestyle advantages and longevity of individuals based on its links in reducing cholesterol levels and blood pressure, attenuating glycaemic response and aiding the establishment of a gut microbiota diversity [36,37]. However, the olive oil industry produces significant levels of by-products, which, whilst high in residual antioxidants, fibre and other associated bioactive co-passengers, are regarded as low value. Hence, the attention to recovering these materials and their molecular compounds has intensified from a pharmaceutical avenue as well as applying them to food systems such as bread and cereal-based foods [38]. This research into the waste minimisation of waste products crosses numerous plant products, such as the work conducted by Romano et al. [34] on the recovery of bioactive compounds from the citrus industry, another high waste-producing manufacturing operation. 

## 3. Refining Extraction Techniques to Enhance Safety and Biological Activities

### 3.1. Food Safety and Environmental Considerations for Waste Recovery

Previous research earlier in this mini-review illustrated the substantial amount of waste that is derived from the by-products and trimmings of fruits, vegetables, grains and even the meat industry. One of the aspects that is commonly observed in food processing is that it often involves the removal of the outer layers of seeds and fruits as well as the core products. This leads to substantial waste production, especially in industries that involve pulping, juicing or refining fruits and vegetables. It needs to be recognised that some of the reasons behind the removal of these components from the edible food streams, as well as the antinutritional/toxic elements, are important to consider when endeavouring to recover as much material as possible from waste streams [8,9]. Bartkiene et al. [39] reported on the potential safety implications when the recovery of food by-products is employed to manufacture food-grade ingredients. What they illustrated was that the choice of extraction technique was crucial in terms of maintaining the safety and functionality of the compounds recovered [40]. Most notably, maintaining the bioactivity and, hence, functionality of these ingredients is a major challenge when considering the efficacy of including these value-added ingredients in food materials. There is also concern in relation to the environmental pressure associated with some extraction processes using harsh chemical treatment in order to recover functional components. For this reason, many researchers have focused on extraction technology, which is often referred to as ‘green’ extraction or processing.

### 3.2. Extraction Processes Which May Be Regarded as Novel

Enzyme-assisted extraction of waste streams has been used as an enzyme that can be used to enhance the release of phenolic compounds from plant matrices, improving extraction efficiency and maintaining the biological structure of the extracted compounds [30,32]. In addition, microbial fermentation of plant waste streams can aid in breaking down complex structures that are prominent in the cellular components of waste streams. Such a disruption to the structure of the cellular components helps in releasing phenolics contained therein. One of the challenges we face is determining the most efficient and cost-effective extraction method for specific plant waste streams, which is essential while ensuring the purity and stability of extracted phenolic compounds for various applications, including food, pharmaceuticals and cosmetics [32]. The establishment of economically feasible processes for phenolic recovery from plant waste streams needs to consider the financial costs associated with extraction and purification, as well as the environmental impact [30,33]. Implementing sustainable practices to minimise the environmental footprint of extraction processes, such as reducing solvent use or utilising ecofriendly methods, is crucial to benefit the environment rather than create more impacts that need mitigating [33,41].

For this reason, there has been increased attention on what could be regarded as novel extraction techniques that rely on cellular disruption of waste materials to aid the overall separation and recovery of functional food components. Notably, Bartkiene et al. [39] found that the combination of ultrasonication and fermentation enhanced the rate of recovery of bioactive ingredients. Speed and ease of recovery of biologically active components useful for the functional food business form an exciting opportunity to speed up the application of food waste streams into food products. Bartkiene et al. [39] also concentrated on ingredient safety, noting that attention still needs to be specific in relation to biogenic amines, mycotoxins and micro- and macroelements. Indeed, contamination of these recovered ingredients can present a number of challenges with heavy metals, residual chemicals and microbial contaminants [42,43]. 

Despite these potential safety issues, the valorisation of the active compounds from agricultural and food processing by-products is important for the future sustainability of the food industry and the upcycling of potentially valuable nutritional compounds [29,34,44]. Lima et al. [45] illustrated that the bioactive compounds extracted from Amazonian fruit and vegetable by-products could be used to significantly change the nutritional profile of foods and thus potentially be used to lower risk factors associated with cancer and metabolic diseases. Phenolic compounds from South American material have been of great interest, as per the research from Beltrán-Medina et al. [46], who have investigated the potential of coffee by-product utilisation in foods, this time including them in extruded snack products and therefore upcycling waste material into a ready-to-eat food product acceptable for consumers. 

Similarly, Park et al. [47] evaluated the potential of spent coffee material as a functional food ingredient and the most economical extraction technique to be used in the recovery of the material for functional food purposes. The researchers illustrated the benefits of sequential extraction techniques and pretreatment methods in enhancing the recovery of high-value products from spent coffee grounds using both supercritical carbon dioxide and ultrasonic treatment. Most notably, ultrasound pretreatment improved the antioxidant activities (DPPH and ABTS assays) of the valorised spent grain compounds into high-value products, illustrating increased efficiency possible through combinations of extraction methods. 

To that end, Tien et al. [48] investigated the efficiency in the recovery of waste material using deep eutectic solvent and sequential microwave-ultrasound-assisted approaches and found that this could be a solution going forward as a method to ensure the breakdown of cellular material and hence the release of bioactive functional compounds from waste products. However, Velusamy, Rajan and Radhakrishnan [49] investigated the use of pulse electric field technology (PEF) as a relatively nonthermal mechanism to break the cellular components of plant walls and enhance the potential of leaching bioactive ingredients during extraction processes. The key themes behind these recent publications are to optimise the recovery rates of beneficial ingredients from waste streams whilst also minimising the impact of the processes employed in the recovery methods. The use of environmental pollutants or high energy-requiring processes flies against the basic tenets of regeneration and sustainability. As mentioned before, this is where computer optimisation and machine learning can help with the green recovery of compounds through the use of technology advancements being created in our development of Industry 4.0 and 5.0 platforms [2,9,14].

### 3.3. Valorised Food Materials and Their Impact on Consumer Health and Wellbeing

One of the common compounds recovered from waste streams in plant-based systems is dietary fibres (and their co-passengers), which are, in turn, beneficial for colonic functionality and gut health [50]. Indeed, the recovery of dietary fibre compounds from by-products has been a constant feature of research interest during the last 5 years [31,51,52,53,54,55,56]. Some fibres act as prebiotics, nourishing beneficial gut bacteria, such as inulin found in chicory root and oligosaccharides in legumes. One premise is that dietary fibres resist digestion in the stomach and small intestine due to resistance to human enzymes. As they reach the large intestine, they become available for fermentation by gut microbiota, which ferment these undigested fibres, breaking them down into short-chain fatty acids (SCFAs) such as acetate, propionate and butyrate. SCFAs support a healthy gut environment by nourishing colon cells, enhancing gut barrier function and regulating pH levels. 

Extending this research into a physiological aspect, these SCFAs influence glucose and lipid metabolism, aiding in blood sugar regulation and reducing cholesterol levels, with further research indicating a potential influence on gene regulation from a cellular basis. Thus, the focus on the recovery of bioactive ingredients that enhance the nutritional quality of foods through enhancing microbial populations and functional gut responses holds the potential for the future [50,53,54]. 

### 3.4. Practical Applications of Isolated Food Components on Consumer Nutrition

There is an issue in terms of how we can ensure that these recovered ingredients can be incorporated into foods, as the enhancements of foods (such as bread and biscuits [53,54,55,56], which are common cereal food that is used in these studies) can affect the texture, structure, mouthfeel and, hence, consumer acceptability of foods using the recovered ingredients, even if the fibre components recovered could be of great help in manipulating gut functionality and chronic diseases, as illustrated by the work of Pansai et al. [57] when recovering fibre compounds from pitya fruit (dragon fruit) and evaluating their mode of improving gut functionality. The work of Hsu, Chang and Shiau [58] is another good example of this, where they endeavoured to recover components from pitya material and incorporate it into bread, finding that the modification of the texture was a challenge to maintain, and whilst it was possible to achieve a product which resembled a control (standard) product, artefacts such as colour and hence taste, also need to be evaluated when incorporating antioxidant-rich, phenolic-containing, substances. This speaks to the requirement for careful attention to consumer preference and how consumer acceptance of novel foods (using these novel ingredients) needs to be considered. 

While Figure 1 illustrates that a major sector for the production of food waste and loss was the fruit and vegetable industries, the meat, fish and dairy sectors are also important to consider. Recently, Rana et al. [59] reviewed the specific potential of fish waste reutilisation in food innovation and suggested a number of products that could be derived from aquatic sources. The FAO [60] has estimated that fish production globally was 214 million tonnes in 2020 and that potentially up to 60% of the weight of the commercial catch is wasted in the form of potential by-products derived from carcass, head, skin and bones [61,62,63,64]. These materials are rich in digestible proteins as well as structural proteins, in addition to minerals and valuable oils. Enzymatic and fermentation processes have been used to recover proteins from fish materials, reducing the use of solvent extraction requirements [62,63]. Such recovered components from fish sources have been used in a wide range of edible food products to the benefit of consumer nutrition and sensory perception, not only in the sense of providing added bioactive components in innovative food products [64,65,66] but also by providing texturizing agents to enhance structure and composition of foods [67,68,69,70].

What the research illustrates is the potential of bioactive compounds—abundant in waste streams of various plant-based products as well as animal sources—that can have significant influences on protein and carbohydrate digestion of novel food products as well as the textural composition for consumer preference. These compounds can lead to the creation of value-added food products from the recovered waste lines [53]. There are many other considerations to be made in relation to regenerative food innovation, such as the utilisation of these resources in clean label ingredients for food processors and exploration of not only the use of the food waste products but material that may be carried over in terms of food processing technologies [71]. Future reviews should focus on these aspects in much more detail and provide a deeper meta-analysis of the key research originating across the world so as to ensure innovation without boundaries occurring in foods. 

## 4. Conclusions

So, where does this leave us in searching for a new way to employ sustainable protocols to create a future for regenerative food innovation? 

Certainly, there exists tremendous potential in using both the phenolic compounds of by-products from food production and processing as well as recovering proven nutritional enhancing components within the bioactive complex that presents itself as dietary fibre and its associated molecules. Implementing efficient production practices, optimising processing techniques to minimise waste generation, and adopting innovative technologies for resource recovery are also of paramount concern. The essential ethos of regenerating is the opportunity to create a new outcome from historical practices. This is where it all gets quite exciting, especially from an academic viewpoint—who are intrinsically connected with applying knowledge for the benefit of society—as we have the potential to use our experience of the past and innovations of the present to develop a future that will relish in the thought development of how to use our vision of technology to create a better future. Thus, redirecting surplus food to food banks, repurposing waste as animal feed or compost, and creating value-added products from by-products has the potential to reduce the overall global impact of waste generation during the production and processing of foods. In order to do this, we need to collaborate across boundaries and disciplines, push our vision to the limits and, if we need to, shake the laws of physics (or science, at least). So, let us enjoy the journey of exploitation and actually utilise the knowledge we have gained from the biological characterisation of the chemical composition of waste materials and the potential new ways of enhancing recovery yields. In order to ensure a true impact is delivered, we need to work in collaboration with industries that are consumer-focused and develop platform products that are acceptable for large-scale consumption. This way, we will move from a pure recovery and utilisation of waste materials to the ability to enhance food innovation systems of the future—regenerative food innovation in practice. 

## Figures and Tables

**Table 1:** Estimated scale of food waste per household on an annual basis in selected countries data obtained from [3].

Country	Total Annual Food Waste (Tonnes)	Food Waste per Capita (kg per Person)
China	91,646,213	64
India	68,760,163	50
United States	19,359,951	59
Japan	8,159,891	64
Germany	6,263,775	75
Australia	2,562,110	102

## Data Availability

The original contributions presented in the study are included in the article, further inquiries can be directed to the corresponding author.
